# Sustained transgene expression from sleeping beauty DNA transposons containing a core fragment of the *HNRPA2B1-CBX3* ubiquitous chromatin opening element (UCOE)

**DOI:** 10.1186/s12896-019-0570-2

**Published:** 2019-11-09

**Authors:** Kristian Alsbjerg Skipper, Anne Kruse Hollensen, Michael N. Antoniou, Jacob Giehm Mikkelsen

**Affiliations:** 1 Department of Biomedicine, HEALTH, Aarhus University, DK- 8000 Aarhus C, Denmark; 2 Department of Molecular Biology and Genetics, Science and Technology, Aarhus University, DK-8000 Aarhus C, Denmark; 3 Gene Expression and Therapy Group, King’s College London, Faculty of Life Sciences & Medicine, Department of Medical and Molecular Genetics, 8th Floor Tower Wing, Guy’s Hospital, London, SE1 9RT UK

## Abstract

**Background:**

DNA transposon-based vectors are effective nonviral tools for gene therapy and genetic engineering of cells. However, promoter DNA methylation and a near-random integration profile, which can result in transgene integration into heterochromatin, renders such vectors vulnerable to transcriptional repression. Therefore, to secure persistent transgene expression it may be necessary to protect transposon-embedded transgenes with anti-transcriptional silencing elements.

**Results:**

We compare four different protective strategies in CHO-K1 cells. Our findings show robust protection from silencing of transgene cassettes mediated by the ubiquitous chromatin-opening element (UCOE) derived from the *HNRPA2B1-CBX3* locus. Using a bioinformatic approach, we define a shorter *HNRPA2B1-CBX3* UCOE core fragment and demonstrate that this can robustly maintain transgene expression after extended passaging of CHO-K1 cells carrying DNA transposon vectors equipped with this protective feature.

**Conclusions:**

Our findings contribute to the understanding of the mechanism of *HNRPA2B1-CBX3* UCOE-based transgene protection and support the use of a correctly oriented core fragment of this UCOE for DNA transposon vector-based production of recombinant proteins in CHO-K1 cells.

## Background

Genomic insertion of transgenes, leading to their stable expression, has been instrumental in studies of gene function and biomedical applications. Stable transgene expression is crucial for a wide range of in vitro experimental setups including disease modelling and production of recombinant proteins. Furthermore, some of the most successful genetic therapies rely on stably integrating and expressing correct copies of disease-causing gene variants [1–3]. Although precise genome editing using the CRISPR/Cas9 system is gaining increasing attention for introducing specific alterations in the genome, ways to achieve stable transgene expression in cell lines, patient cells, or tissues remain essential for many purposes including long-term therapeutic efficacy of cell and gene therapies.

In order to ensure reliable, long-lasting transgene expression, several *cis*-acting elements have been utilized and included in the design of gene transfer vectors [4]. These elements act by shielding the transgene cassette from position effect variegation (PEV), thereby avoiding the spread of heterochromatin and hypermethylation into the integrated transgene cassette. Until now, several different protective elements, including the 5’HS chicken β-globin (cHS4) insulator [5], the D4Z4 insulator [6] and Ubiquitous Chromatin Opening Elements (UCOE) [7, 8] have been exploited for protection of transgenes. The cHS4 insulator, widely used in the context of integrating viral and nonviral vectors [9–14], functions by blocking enhancer activity and, when flanking the transgene, by acting as a barrier against PEV [5, 15]. Interestingly, several DNA-binding proteins are recruited to the cHS4 insulator. Enhancer-blocking activity has been attributed to the CCCTC-binding factor (CTCF) [16, 17], whereas Upstream Stimulatory Factors 1 and 2 (USF 1 and 2) and Poly(ADP-ribose) Polymerase-1 (PARP-1) are thought to confer barrier activity of the insulator [18–20]. The UCOE sequence derived from a human CpG island encompassing the bidirectional promoters driving expression of Chromobox Protein Homolog 3 (CBX3) and Heterogeneous Nuclear Ribonucleoproteins A2/B1 (together referred to as the *HNRPA2B1-CBX3* locus), has also been extensively studied for protection against transcriptional silencing [21–24]. The element was initially shown to confer stable enhanced green fluorescent protein (eGFP) expression when integrated as part of the transgene expression cassette into centromeric heterochromatin [7]. Since then, several different fragments derived from the *HNRPA2B1-CBX3* locus have been utilized in various gene vehicles with a 1.5 kb fragment (1.5UCOE) as the most frequently used variant [21–23, 25–27]. Additional file 1: Figure S1 provides an overview of different UCOE fragments that have been used. In contrast to cHS4, for which insulating abilities have been attributed to the recruitment of several DNA-binding proteins, the UCOE mechanism of action is still poorly understood. The endogenous locus has been found to be hypomethylated in peripheral blood mononuclear cells [28], and different UCOE fragments in viral vectors have been shown to confer both hypomethylation and enrichment of the permissive histone H3K4 trimethylation (H3K4me3) mark [22, 27]. Furthermore, areas with high CpG density in the CBX3 region have recently been shown to be critical for conferring UCOE function and protection against transgene silencing [22, 29]. Nevertheless, whether a high CpG density in itself confers the anti-silencing function of the UCOE or whether this UCOE recruits DNA-binding proteins aiding in protecting transgenes, as has been described for both cHS4 and D4Z4 insulators [16, 18, 30], remains to be determined, although involvement of CTCF and CXXC finger protein (CFP1) has been suggested [27].

We have previously utilized the cHS4 insulator to shield gene cassettes in *sleeping beauty* (SB) transposon vectors to mediate protection against transgene silencing in F9 murine teratocarcinoma cells [31]. We also found increased vector mobilization when including protective *cis*-acting elements into DNA transposon vectors [32]. In the present study, we embarked on an investigation to assess UCOE-directed protection of transgene silencing in the context of SB DNA transposon vectors introduced into Chinese hamster ovary (CHO) K1 cells, which are widely used for the industrial production of recombinant proteins from chromosomally integrated transgenes [33]. Using an eGFP reporter gene, we showed rapid and robust repression of expression with transgenes driven by the CMV promoter alone. In constructs where the CMV promoter was linked with either cHS4, D4Z4 or 1.5 kb *HNRPA2B1-CBX3* UCOE (1.5UCOE), we observed reduced silencing with both cHS4- and UCOE-protected DNA transposons. Based on a bioinformatic approach, we identified a core fragment within the 1.5UCOE and demonstrated that this element can confer effective protection from transcriptional silencing upon SB DNA transposon-based gene transfer.

## Results

### Comparison of protective elements in the context of sleeping beauty DNA transposon vectors

**Fig. 1:**
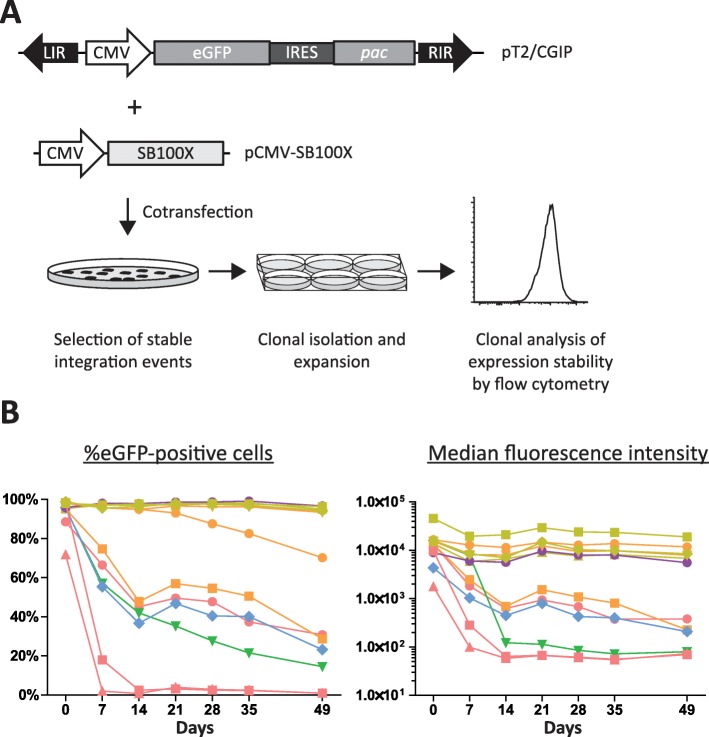
Progressive silencing of *Sleeping Beauty* transposon vectors in CHO-K1 cells. **a** Illustration of the experimental procedure used in this study including a schematic representation of the SB vector construct. **b** Evaluation of stability of transgene expression in single cell CHO-K1 clones containing SB transposon vectors. On Day 0, puromycin was removed from the medium and eGFP expression monitored by flow cytometry at different time points for 7 weeks. Both the total percentage of eGFP-positive cells and the median fluorescence intensity is shown. Each line in the graphs represents the expression profile of a single clone over the course of the 7 weeks of continuous culture. LIR: Left inverted repeat, RIR: Right inverted repeat, CMV: Cytomegalovirus promoter, eGFP: enhanced Green fluorescent protein, IRES: Internal ribosomal entry site, pac: puromycin N-acetyl-transferase

The SB DNA transposon system has become a powerful tool for mediating integration into the genome of target cells by means that do not involve virus-based gene transfer. As such, the system has been widely used for production of cell lines stably expressing transgenes of interest [34–36]. One of the key advantages of the SB transposon is its near random integration profile [37, 38], minimizing the risk of deleterious integrations. However, this increases the possibility of transgene integration into condensed (constitutive or facultative) heterochromatin, thereby leaving the integrated transposon cassette more vulnerable to transcriptional repression. In addition, even transgene integration events within transcriptionally permissive euchromatic regions can still be silenced by promoter DNA methylation. Reliable use of DNA transposon-based vectors may thus require inclusion of genetic elements that protect the inserted transgene against silencing. We therefore investigated the shielding of DNA transposon-embedded gene cassettes in CHO-K1 cells, by exploiting a model system for monitoring transgene stability following transposon-mediated integration. Similar to an approach that we previously described [32], we constructed a SB transposon vector, pT2/CGIP, carrying a CMV-driven eGFP-IRES-*pac* (CGIP) cassette conferring expression of both eGFP and puromycin N-acetyltransferase (pac) (Fig. 1a). This enabled us to not only monitor transgene expression over time by flow cytometry, but also to maintain stable expression by keeping CHO-K1 clones under continuous selection with puromycin. We co-transfected CHO-K1 cells with the pT2/CGIP vector together with a plasmid encoding either the hyperactive SB transposase SB100X or an inactive mutant variant (mSB). SB-mediated integration was confirmed by counting the number of puromycin-resistant colonies obtained with the SB100X and mSB transposase variants (Additional file 1: Figure S2). An overview of the experimental workflow is shown in Fig. 1a. A total of twelve clones harboring SB100X-directed transgene insertions were then passaged for 7 weeks in the absence of puromycin, and eGFP expression was monitored weekly by flow cytometry (Fig. 1b, Additional file 1: Figure S3). With six of the clones we observed a rapid decrease in eGFP expression even by 7 days following removal of puromycin selection pressure. In addition, in one clone, which initially appeared stable, we observed from day-21 to 49 a gradual decrease in the percentage of eGFP-positive cells (93 to 70%). Notably, the median fluorescence intensity (MFI) for this clone remained stable throughout the seven-week period, suggesting that expression of eGFP was only slowly silenced and that prolonged passage was required to measure an MFI decrease in this clone.

**Fig. 2:**
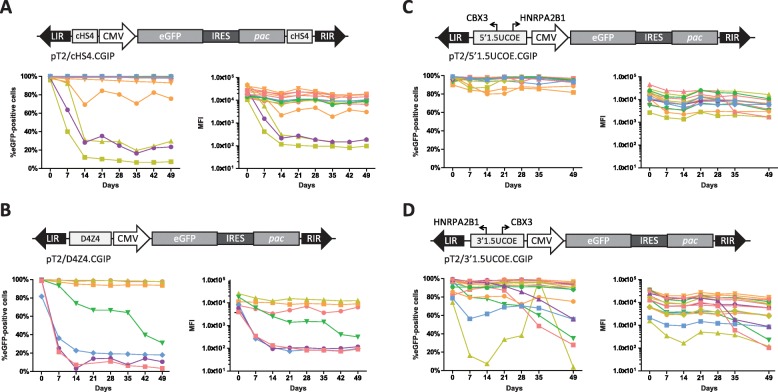
Robust protection from transcriptional silencing mediated by cHS4 and 1.5UCOE elements in *Sleeping Beauty* transposon vectors. Schematic illustration of SB transposon vector constructs and analysis of their respective analysis of eGFP expression in single CHO-K1 clones over a 7 week period of continuous culture in the absence of puromycin selective pressure with either cHS4 (**a**), D4Z4 (**b**) or the 1.5UCOE in a 5′- (**c**) or 3′- (**d**) orientation. Each line in the graphs represents the expression profile of a single clone over the course of the 7 weeks of continuous culture

Having established that the CMV-driven eGFP cassette was frequently repressed in CHO-K1 cells as previously reported [21], we moved on to investigate the effect of including three different *cis*-acting elements, which have been reported to confer protection against epigenetic-mediated silencing, namely a 1.5 kb fragment of the *HNRPA2B1-CBX3* UCOE (1.5UCOE) [19–22], cHS4 [9, 11, 32] and D4Z4 [30] in the SB transposon vector. The different elements were cloned into pT2/CGIP (Fig. 2a-d). The 1.5UCOE and D4Z4 elements were placed immediately upstream from the CMV promoter. As the 1.5UCOE has been shown to work both in the 5′ -and 3′-orientation [21, 25] vectors carrying the element inserted in both orientations were constructed and tested. As the cHS4 insulator only displays anti-transcriptional silencing by flanking transgenes [9, 11, 32], we inserted copies of the 1.2-kb version of this insulator element both upstream and downstream from the expression cassette. The vectors were used to generate puromycin-resistant CHO-K1 clones using SB100X (Fig. 1a), and a total of 51 clones (14 with T2/5′1.5UCOE.CGIP, 16 with T2/3′1.5UCOE.CGIP, 14 with T2/cHS4.CGIP, and 7 with T2/D4Z4.CGIP) were isolated and expanded. We first analyzed the level of eGFP expression in puromycin-selected clones and as expected observed substantial interclonal differences (Additional file 1: Figure S4), most likely due to copy number variation and position effects affecting gene expression. Relative to clones carrying the unprotected CGIP cassette, the 1.5UCOE did not in either of the two orientations increase eGFP expression, in contrast to previous reports [7, 21], whereas cHS4 insulators flanking the CGIP cassette both increased mean expression levels and lowered the interclonal variation, in line with previous studies (Table 1, Additional file 1: Figure S4) [39, 40].

**Table 1:** Overview of the studied protective elements and their ability to sustain transgene expression in CHO-K1 cells

Group	Element size (kb)	No. of clones analyzed	Day 0 MFI^a^	Day 0 interclonal variation^a^	Day 0:Day 49 eGFP ratio^a^	Stable clones (%)^b^
CGIP	–	12	14,549 ± 0.3129	8.238 ± 1.772	0.56 ± 0.12	42
cHS4	2 × 1.2	14	24,838 ± 0.3454	2.559 ± 0.3559	0.79 ± 0.16	71
D4Z4	3.3	7	11,265 ± 0.2904	3.035 ± 0.7825	0.56 ± 0.10	43
5’1.5UCOE	1.5	14	17,014 ± 0.2988	6.358 ± 1.117	0.97 ± 0.01	100
3’1.5UCOE	1.5	16	17,085 ± 0.3009	10.97 ± 1.932	0.78 ± 0.08	63
5’UCOE-CORE	0.86	13	20,498 ± 0.5042	4.790 ± 1.178	0.46 ± 0.11	31
3’UCOE-CORE	0.86	13	20,256 ± 0.3585	4.454 ± 0.7882	0.87 ± 0.08	77

^a^mean ± SEM, ^b^ eGFP ratio ≥ 0.8

To evaluate the stability of expression over time, we then passaged the clones for 7 weeks in the absence of puromycin and monitored eGFP expression weekly by flow cytometry. Among the 14 clones harboring cHS4-flanked CGIP, only 4 clones gradually lost expression, validating the protective effect of cHS4 in CHO-K1 cells (Fig. 2a, Additional file 1: Figure S3). The D4Z4 insulator, in contrast, did not appear to negate transcriptional silencing with 4 out of 7 clones being progressively silenced (Fig. 2b, Additional file 1: Figure S3). Notably, loss of expression was not observed in any of the 14 clones carrying T2/5′1.5UCOE.CGIP (Fig. 2c, Additional file 1: Figure S3), demonstrating a robust capability of the 1.5UCOE element to protect against silencing resulting in negligible interclonal variation. Among the 16 clones harboring CGIP with the 1.5UCOE in the 3′ orientation at least 6 clones gradually lost eGFP expression during passaging resulting in substantial variation between the clones (Fig. 2d, Additional file 1: Figure S3). Nevertheless, the majority of clones harboring T2/3′1.5UCOE.CGIP remained stable throughout the experiment, indicating that although minor direction-dependent differences were seen, 1.5UCOE efficiently protected against silencing in both orientations. Interestingly, this is in contrast to a previous study conducted in murine P19 embryonal carcinoma cells [26], in which UCOE function relied heavily on the orientation of the element with the 3′-orientation appearing to confer most robust protection. As the *HNRPA2B1-CBX3* UCOE encompasses two divergently transcribing promotors, it is plausible that transcriptional activation by these promoters is crucial for and even dictates the protective function by this element. In summary, our results demonstrate that both 1.5UCOE and the cHS4 insulator elements, but not the D4Z4 insulator, is able to shield SB transposon vectors and protect against transgene silencing in the CHO-K1 cell line. Due to protective properties and small size of 1.5UCOE, we focused on optimizing this element as a protective add-on to DNA transposon vectors used in CHO-K1 cells.

### Bioinformatic analysis of the endogenous HNRPA2B1-CBX3 locus reveals extensive transcription factor binding

**Fig. 3:**
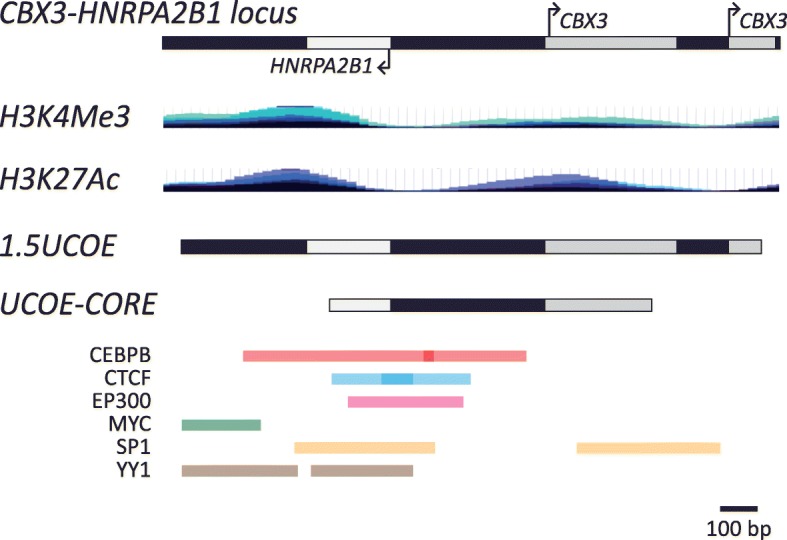
Several transcription factors can be recruited to the endogenous *CBX3-HNRPA2B1* locus and may regulate UCOE function. Schematic illustration of the endogenous *CBX3-HNRPA2B1* locus with layered H3K4Me3 and H3K27Ac ChIP-seq tracks from the ENCODE project and overview of conserved transcription factor binding sites from ConTra v3 overlapping with ENCODE transcription factor ChIP-seq peaks. Each bar represents an overlapping region from a specific transcription factor (CEBPB: red, CTCF: blue, EP300: pink, MYC: green, SP1: yellow, YY1: brown). The location of the 1.5 kb *HNRPA2B1-CBX3* UCOE region (1.5UCOE) is indicated as is the derived UCOE-CORE. Scale bar: 100 bp

In order to further investigate the mechanism underpinning the transcriptional protective capability of the *HNRPA2B1-CBX3* UCOE, we utilized a bioinformatic approach to map transcription factor binding sites in this element. To identify potential key regulators of UCOE function, we combined data from the ENCODE project with an analysis of protein-protein interactions and conserved transcription factor binding motifs. A total of 89 proteins were found to be potentially associated with the endogenous *HNRPA2B1-CBX3* locus using ChIP-seq data from the ENCODE project (see Additional file 2 for a list of proteins with identified peaks within the locus). In line with the status of both *CBX3* and *HNRPA2B1* as constitutively transcribed housekeeping genes, a large fraction of the identified proteins was associated with the RNA polymerase II transcription machinery and therefore not directly relevant for identifying key UCOE regulatory sites. Instead, based on reported roles in modulating chromatin structure and/or insulator function, we identified a subset of transcription factors (Fig. 3), for which enriched Gene Ontology (GO) terms are provided in Additional file 1: Figure S5. We identified physical protein-protein interactions by utilizing the BioGRID database together with esyN [41, 42] (Additional file 1: Figure S6) and identified a major cluster of potentially interacting transcription factors. E1A binding protein p300 (EP300) and SP1 had the highest number of interactions, six and five interactors, respectively, suggesting a role of these proteins in UCOE function. The two transcription factors, HMGN3 and CHD2, were not found to have any interactions within the cluster (Additional file 1: Figure S6). To strengthen our predictions, ConTra V3 [43] was utilized to identify predicted conserved transcription factor binding motifs, which were then cross-referenced with the ENCODE ChIP-seq data (Fig. 3). Several transcription factors showed significant overlap between the BioGRID database and the ChIP-seq data sets. Notably, SP1 and EP300 were again found to be prominent in this analysis emphasizing their potential recruitment to the locus and key role in UCOE function (Fig. 3, pink and yellow bars). Yin Yang 1 (YY1), a transcription factor described to be recruited to a methylation-sensitive insulator [44], was also identified. Intriguingly, together with SP1 binding motifs YY1 binding sites have been found to be enriched in bidirectional promoter loci compared to unidirectional promoter loci [45], supporting their potential recruitment to this UCOE locus. Furthermore, conserved binding motifs for CTCF were also found to overlap with ChIP-seq peaks. CTCF is associated with the function of methylation-determining regions [46] and is also responsible for mediating enhancer-blocking activity of the cHS4 insulator [16, 17]. CEBPB and MYC were also identified as potential players in UCOE function in this analysis. Based on these predictions, we deduced an 863-bp ‘core’ fragment within the *HNRPA2B1-CBX3* UCOE (UCOE-CORE) containing the motifs necessary to recruit and bind transcription factors to this UCOE and to maintain an open chromatin environment (Fig. 3).

### Robust orientation-dependent protection by the UCOE-CORE element in DNA transposon vectors integrated into CHO-K1 cells

**Fig. 4:**
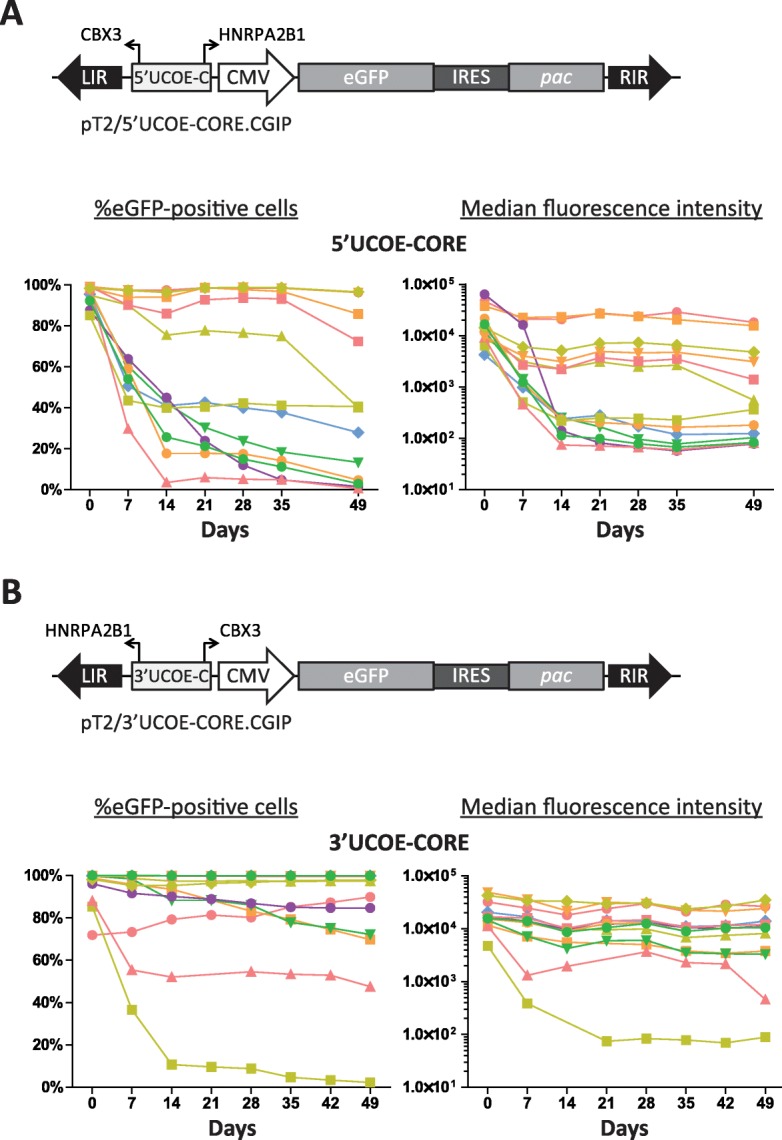
UCOE-CORE element provides direction-dependent, robust protection against transgene repression. Schematic illustration of the SB transposon vectors carrying the UCOE-CORE element and eGFP expression profile of individual CHO-K1 clones harboring these vectors over a seven-week period of continuous culture in the absence of puromycin selection pressure with the UCOE-CORE in either the 5′-(**a**) or 3′- (**b**) orientation. Expression of eGFP was assessed by flow cytometry. Each line in the graphs represents the expression profile of a single clone over the course of the 7 weeks of continuous culture

For expression vectors to accommodate promoters and genes of a considerable size, inclusion of silencing protective elements of restricted size is attractive and indeed may be necessary. In order to test the capability of the UCOE-CORE to protect a DNA-transposon-delivered CGIP cassette, the 863-bp fragment was inserted 5′ of the CMV promoter in the pT2/CGIP vector in both 5′ -and 3′-orientations (Fig. 4a, b, pT2/5’UCOE-CORE.CGIP and pT2/3’UCOE-CORE.CGIP). These were then stably transposed into CHO-K1 cells using SB100X and a total of 26 clones isolated and eGFP expression followed over time. As in the case of the 1.5UCOE element, inclusion of the UCOE-CORE did not appear to increase initial eGFP expression levels (Table 1, Additional file 1: Figure S7). However, as predicted by the transcription factor binding site analysis (Fig. 3), the UCOE-CORE was capable of negating transcriptional repression in the CHO-K1 cell line. Notably, in contrast to the 1.5UCOE element, which showed little dependence on orientation for function (Fig. 2c, d), the UCOE-CORE element displayed a strong directional bias. We observed that 10 out of 13 clones with the 5’UCOE-CORE-CGIP cassette were progressively silenced after removal of puromycin during the period 7 weeks of continuous culture (Fig. 4a, Additional file 1: Figure S8). In marked contrast, in clones harboring constructs with the UCOE-CORE element placed in the 3′-orientation; that is, with the *CBX3* promoter in the sense direction, only 3 out of 13 clones showed a gradual loss in eGFP reporter gene expression over the same time course (Fig. 4b, Additional file 1: Figure S8). Thus, the 3’UCOE-CORE performed at a level comparable to that of the cHS4 insulator and the 1.5UCOE (Table 1). In addition, prolonged passage of the clones carrying the 3’UCOE-CORE element resulted in little change in eGFP expression levels over 100 days of continuous culture, demonstrating the robustness of the element in conferring stability of function (Additional file 1: Figure S9).

## Discussion

Although different *HNRPA2B1-CBX3* UCOE fragments have been extensively utilized for mediating stable gene expression, little is known about the mechanism of action of this element. *HNRPA2B1-CBX3* UCOE fragments have previously been shown to confer both hypomethylation and enrichment of H3K4me3 marks [22, 27]. In addition, the presence of an methylation-determining region [46] downstream of the *CBX3* promoter within the 2.2UCOE has been proposed [22]. The existence of several transcription factors that may potentially bind to the *HNRPA2B1-CBX3* UCOE and influence its function (Fig. 3), many of which are associated with chromatin remodeling, may suggest a possible link between the UCOE sequence and its ability to model chromatin. The location of the predicted transcription factor binding sites indicated the presence of a core region located between the transcriptional start sites of *HNRPA2B1* and *CBX3* generating an open chromatin environment. Indeed, when we tested this predicted core as a *cis*-acting element, we observed robust eGFP expression up to 100 days after removal of puromycin selective pressure (Fig. 4; Additional file 1: Figure S9).

Reports showing the anti-silencing capacity of several non-overlapping *HNRPA2B1-CBX3* UCOE fragments (Additional file 1: Figure S1) [21–23, 26, 27, 47], indicate that this element contains several functional subregions, all with the ability to generate an epigenetically, transcriptionally permissive environment. Whether these regions are completely independent or can work in concert to secure stable expression is not known. Of potential note is that the 3’UCOE-CORE element, which possesses both *HNRPA2B1* and *CBX3* promoters appears to provide somewhat greater stability of expression (Fig. 4b) than fragments encompassing just the *CBX3* half of the 1.5UCOE [22, 27, 48]. This suggests that the dual divergently transcribed promoter structure of the UCOE-CORE element may confer a more potent anti-transcriptional silencing capability.

In our study we found a bias related to the orientation of a *HNRPA2B1-CBX3* UCOE core segment that has not previously not been reported. Interestingly, this orientation bias appeared when parts of *HNRPA2B1* and *CBX3* were removed, indicating that one or more regulatory regions within either of these deleted fragments, necessary to ensure stable expression in the 5′-orientation, were lost in the UCOE-CORE element (Fig. 2c, d, 4). Indeed, the identification of functional elements located outside of the UCOE-CORE sequence that we defined in our studies, suggests that such additional regulatory sites exist [22, 47]. Interestingly, a direction-dependent function of the *HNRPA2B1-CBX3* UCOE has been described in P19 cells for several different UCOE fragments [7, 22, 26]. The underlying cause for this apparent bias remains unknown. Nevertheless, it is possible that the overall structure of the *HNRPA2B1-CBX3* UCOE, encompassing two divergently transcribed promoters, plays a role in determining its function, as independent and differential expression from the *CBX3* and *HNRPA2B1* promoters may involve a direction-dependent function. Studies of *CBX5-HNRNPA1*, a ‘sister’ locus to *HNRPA2B1*-*CBX3*, have previously demonstrated independent transcription from two divergently transcribed promoters [49–51]. It is thus feasible that utilizing the *HNRPA2B1-CBX3* UCOE in a cell line with either low endogenous *CBX3* or *HNRPA2B1* expression can create a direction-dependent function, underlining the necessity of testing this element in both orientations when utilizing this UCOE in a new cellular context.

In summary, our studies compare the functionality of four different SB-based vectors containing protective elements for stable long-term expression in CHO-K1 cells. We found that the cHS4 insulator and 1.5UCOE possessed an equal anti-transcriptional silencing capability. However, unlike the cHS4 insulator that must be inserted at both 5′ and 3′ ends of a transgene to confer transcriptional stability, the 1.5UCOE and its smaller 863 bp 3’UCOE-CORE are fully functional as a single element placed upstream of the heterologous promoter driving expression of the gene of interest. Thus, the use of these UCOE elements, especially the 3’UCOE-CORE offers a significant space saving advantage over the cHS4 insulators that must be repeated at both ends of the transposon transgene cassette. In addition, the use of a single 3’UCOE-CORE also reduces propensity for transgene loss through recombination as may occur between insulators that that flank both ends of the transposon transgene. The data we present also lays the foundation for further investigations into the role of transcription factors associated with *HNRPA2B1-CBX3* UCOE-containing transgene cassettes. However, our initial attempts to generate CHO-K1 cells carrying CRISPR-derived knockout mutations restricting Sp1 and CTCF expression led to extensive cell death, suggesting that these factors are essential for cell growth and survival. Although this may complicate studies focusing on these genes, knockdown approaches based on CRISPRi or RNA interference may allow investigations of the impact of Sp1 and CTCF on UCOE function. Our data showed robustness of a the UCOE-CORE segment in the context of SB DNA transposon vectors, resulting in maintained transgene expression even after extended continuous cell culture. These findings define a short UCOE variant suitable for transgene expression purposes and provide a platform for functional analysis of UCOE action.

## Conclusions

In conclusion, the results presented in this study contribute to the understanding of the mechanisms of action of the *HNRPA2B1-CBX3* UCOE and its use for protection against transgene silencing thereby laying the groundwork for improved vector design. Our data presented here furthermore stress the importance of including a protective *cis*-element, like the UCOE-CORE segment, in integrated DNA transposon vectors used for production of recombinant proteins in CHO-K1 and similar cell lines.

## Methods

### Plasmid construction

All plasmid constructions were done in pT2/CMV-eGFP(s).SV40-neo [52] containing a second-generation SB transposon backbone. Initially, the CMV-eGFP(s).SV40-neo fragment was replaced with a polylinker containing multiple restriction enzyme sites to expedite insertion of DNA fragments and cloning by HindIII digestion of the pT2 backbone and insertion of annealed double-stranded oligonucleotides with compatible overhangs; this created the construct pT2/Linker. This vector was then used to generate pT2/CMV-eGFP by insertion of the EcoRI-excised CMV-eGFP expression cassette from pT2/CMV-eGFP(s).SV40-neo into EcoRI-digested pT2/Linker. The 1.5UCOE element is a 1.5 kb Esp3I genomic fragment is derived from the *HNRPA2B1-CBX3* housekeeping gene region, extending over the transcriptional start sites of these two divergently transcribed genes [21]. The 1.5UCOE was isolated from a pBluescript subclone (MA895) of this region by PCR amplification and inserted into AvrII-digested and Klenow-treated pT2/CMV-eGFP by blunt-end ligation. Flanking the CMV-eGFP expression cassette with cHS4 was done by insertion of either AvrII -or AgeI-digested cHS4 from the pSBT/cHS4-PGK-Puro-cHS4 [31] upstream or downstream of the cassette, respectively. pT2/D4Z4.CMV-eGFP was created by insertion of a KpnI-digested D4Z4 fragment from C1X [6] into KpnI-digested pT2/CMV-eGFP. Finally, an IRES-*pac-*pA fragment was inserted into the pT2 vectors by PCR amplification from the pSBT/CMV-eGFP-IRES-*pac* and subsequent insertion into PacI -and AgeI-digested pT2 vectors to create pT2/CGIP, pT2/5′1.5UCOE.CGIP, pT2/3′1.5UCOE.CGIP, pT2/cHS4.CGIP and pT2/D4Z4.CGIP. pT2/5’UCOE-CORE.CGIP and pT2/3’UCOE-CORE.CGIP were created by insertion of the PCR-amplified UCOE-CORE fragment from pT2/5′1.5UCOE.CGIP into a EcoRI-digested pT2/CGIP backbone. pCMV-SB100X has been previously described [53]. UCOE is an official registered trademark (Merck MilliporeSigma).

### Cell culture and transfections

CHO-K1 were a kindly provided by professor Per Höllsberg, Department of Biomedicine, Aarhus University. The cells were cultured at 37 °C in a 5% (v/v) CO_2_ atmosphere. The cells were maintained in Dulbecco’s Modified Eagle’s Medium (DMEM) (Sigma-Aldrich, St. Louis, MO, USA) supplemented with 5% fetal calf serum, 100 U/ml penicillin and 100 μg/ml streptomycin. For transfections, 2.5 × 10^5^ cells were seeded in 6-well plates and the following day transfected with 250 ng of the hyperactive SB transposase-expressing plasmid pCMV-SB100X together with 250 ng of either pT2/CGIP, pT2/5′1.5UCOE.CGIP, pT2/3′1.5UCOE.CGIP, pT2/cHS4.CGIP, pT2/D4Z4.CGIP, pT2/5’UCOE-CORE.CGIP or pT2/3’UCOE-CORE.CGIP in a 1:1 ratio using the TurboFect transfection reagent (Thermo Fisher Scientific, Waltham, MA, USA) according to manufacturer’s instructions. For quantification of colony formation, the cells were then reseeded in P10 Petri dishes in appropriate dilutions and the medium was subsequently replaced with antibiotic selection medium containing 5 μg/ml puromycin. After 14 days of passage, the cells were fixed and stained with a 0.6% methylene blue solution, and colonies counted. For isolation and expansion of single clones, the cells were diluted 1:500 and reseeded in a 96-well plate the day after transfection. The cells were maintained in antibiotic selection medium as above, and resistant clones were expanded for analysis. All the resulting clones were kept in antibiotic selection medium unless otherwise specified.

### Bioinformatics

Analysis of ChIP-seq data from the ENCODE project [54] was done using the built-in track on the UCSC Genome Browser, GRCh37/hg19 human genome assembly. All transcription factors showing a peak of binding activity within the 1.5UCOE region (chr7:26,239,852-26,241,407) were included. For analysis of physical protein-protein interactions, a subset of transcription factors were chosen based on current literature and analyzed using the esyN network builder [41] coupled with the BioGRID database v3.5.165 [42]. Finally, the ConTra v3 [43] web server was utilized to identify conserved transcription factor binding sites using all available databases and the following stringency settings; core = 0.95, similarity matrix = 0.85. These were then loaded into the UCSC Genome Browser to cross-reference with the ENCODE ChIP-seq data tracks.

### Flow cytometry

CHO-K1 clones were trypsinized and centrifuged at 500 g for 3 min at 4 °C, washed in Dulbecco’s Phosphate Buffered Saline (DPBS) and finally resuspended in 250 μl DPBS for analysis in 96-well plates. Flow cytometry analysis was carried out at the FACS Core Facility, Aarhus University, on a BD LSRFortessa Cell Analyzer (Becton Dickinson, Franklin Lakes, NJ, USA) with a BD High Throughput Sampler. Data analysis was performed using FlowJo (v.10.4, FlowJo, LLC, Ashland, OR, USA) and the gating strategy can be seen in, Additional file 1: Figure S10.

## Supplementary information

**Additional file 1: Figure S1.** Schematic illustration of the endogenous CBX3-HNRPA2B1 locus with the different UCOE fragments indicated. **Figure S2.** Sleeping Beauty-mediated colony formation in CHO-K1 cells. Data is presented as mean ± SEM and *n* = 3. **Figure S3.** Flow cytometric analysis of representative clones within each group. **Figure S4.** Construct-dependent clonal variation in eGFP expression levels. eGFP-expression MFIs for the clones in each group were normalized to the lowest expressing clone in the individual groups. Boxes are displayed as Q2 + Q3 quantile, and whiskers show 10–90 percentile. **Figure S5.** Top ten enriched GO terms for the selected subset. **Figure S6.** esyN protein-protein interaction network for the selected subset of transcription factors. The size difference indicates the most central nodes as calculated by the betweenness centrality of each node. **Figure S7.** Clonal variation in eGFP expression levels in UCOE-CORE clones. eGFP-expression MFIs for the clones in each group were normalized to the lowest expressing clone in the individual groups. Boxes are displayed as Q2 + Q3 quantile, and whiskers show 10–90 percentile. **Figure S8.** Flow cytometric analysis of representative clones harbouring either the 5’UCOE-CORE or the 3’UCOE-CORE. **Figure S9.** Extended analysis of clones containing the 3’UCOE-CORE. Days 0–49 correspond to data presented in Fig. 4b. **Figure S10.** Flow cytometry gating strategy.

**Additional file 2. **List of proteins with identified ChIP-seq peaks from the ENCODE project within the *CBX3-HNRPA2B1* locus.

## Data Availability

The datasets generated during the current study are available in the Harvard Dataverse repository, 10.7910/DVN/MIHA3Q.

## Abbreviations

CBX3: Chromobox Protein Homolog 3
CFP1: CXXC finger protein
CHO: Chinese hamster ovary
cHS4: 5’HS chicken β-globin (cHS4) insulator
CTCF: CCCTC-binding factor
eGFP: Enhanced green fluorescent protein
EP300: E1A binding protein p300
GO: Gene ontology
H3K4me3: H3K4 trimethylation
HNRPA2B1: Heterogeneous Nuclear Ribonucleoproteins A2/B1
MFI: Median fluorescence intensity
PAC: Puromycin N-acetyltransferase
PARP-1: Poly(ADP-ribose) Polymerase-1
PEV: Position effect variegation
SB: Sleeping Beauty
UCOE: Ubiquitous chromatin-opening element
USF1: Upstream Stimulatory Factor 1
USF2: Upstream Stimulatory Factor 2
YY1: Yin Yang 1
