# Supplementary material for: Sustained transgene expression from sleeping beauty DNA transposons containing a core fragment of the HNRPA2B1-CBX3 ubiquitous chromatin opening element (UCOE)
Source: BMC Biotechnol. 2019 Nov 9;19:75. doi: 10.1186/s12896-019-0570-2 (PMC6842454; doi:10.1186/s12896-019-0570-2)
Supplement: Supplementary file 1 — Additional file 1: Figure S1. Schematic illustration of the endogenous CBX3-HNRPA2B1 locus with the different UCOE fragments indicated. Figure S2. Sleeping Beauty-mediated colony formation in CHO-K1 cells. Data is presented as mean ± SEM and n = 3. Figure S3. Flow cytometric analysis of representative clones within each group. Figure S4. Construct-dependent clonal variation in eGFP expression levels. eGFP-expression MFIs for the clones in each group were normalized to the lowest expressing clone in the individual groups. Boxes are displayed as Q2 + Q3 quantile, and whiskers show 10–90 percentile. Figure S5. Top ten enriched GO terms for the selected subset. Figure S6. esyN protein-protein interaction network for the selected subset of transcription factors. The size difference indicates the most central nodes as calculated by the betweenness centrality of each node. Figure S7. Clonal variation in eGFP expression levels in UCOE-CORE clones. eGFP-expression MFIs for the clones in each group were normalized to the lowest expressing clone in the individual groups. Boxes are displayed as Q2 + Q3 quantile, and whiskers show 10–90 percentile. Figure S8. Flow cytometric analysis of representative clones harbouring either the 5’UCOE-CORE or the 3’UCOE-CORE. Figure S9. Extended analysis of clones containing the 3’UCOE-CORE. Days 0–49 correspond to data presented in Fig. 4b. Figure S10. Flow cytometry gating strategy. [file 12896_2019_570_MOESM1_ESM.pdf]

Additional file 1

Sustained transgene expression from Sleeping Beauty DNA transposons containing a core fragment of the HNRPA2B1-CBX3 ubiquitous chromatin opening element (UCOE)

Kristian Alsbjerg Skipper, Anne Kruse Hollensen, Michael N. Antoniou and Jacob Giehm Mikkelsen

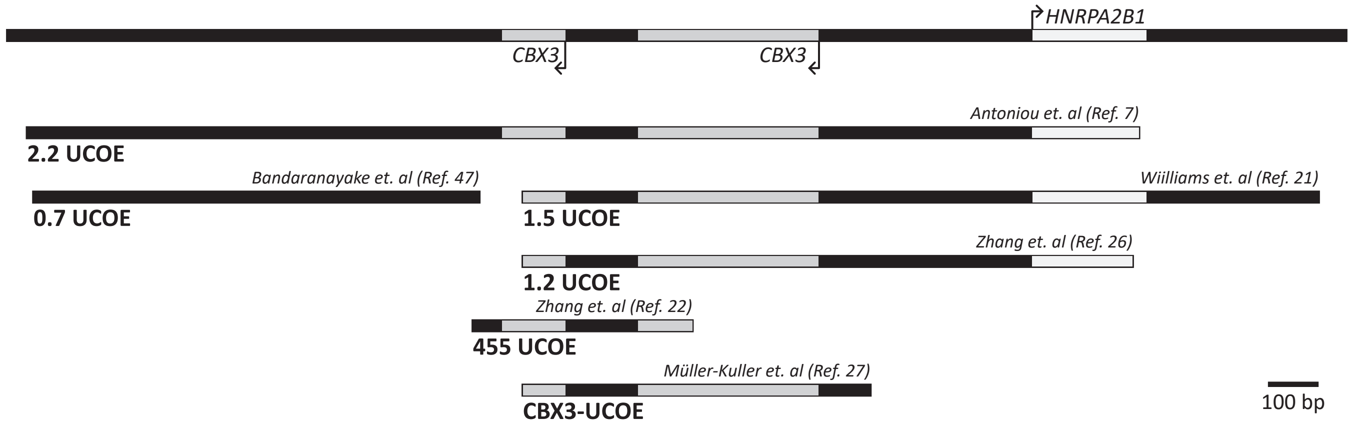

Figure S1. Schematic illustration of the endogenous CBX3-HNRPA2B1 locus with the different UCOE fragments indicated.

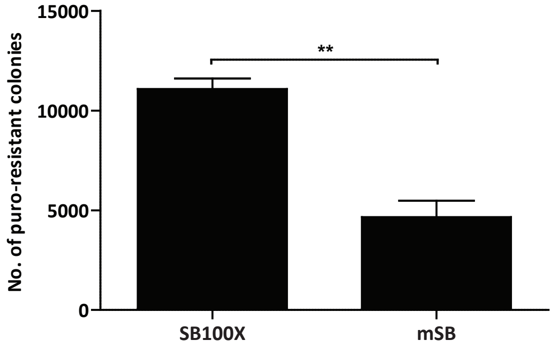

Figure S2. Sleeping Beauty-mediated colony formation in CHO-K1 cells. Data is presented as mean ± SEM and n = 3.

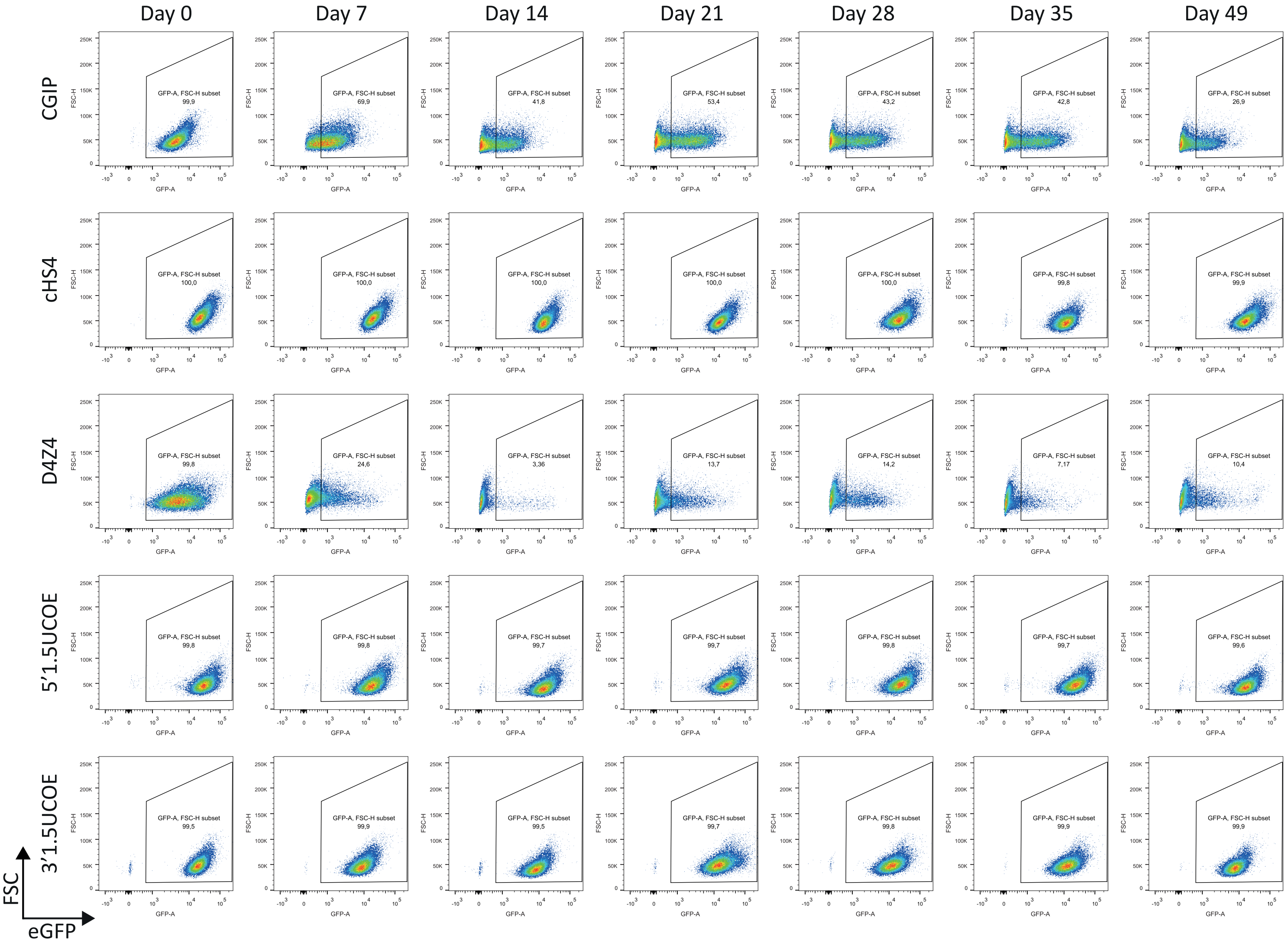

Figure S3. Flow cytometric analysis of representative clones within each group.

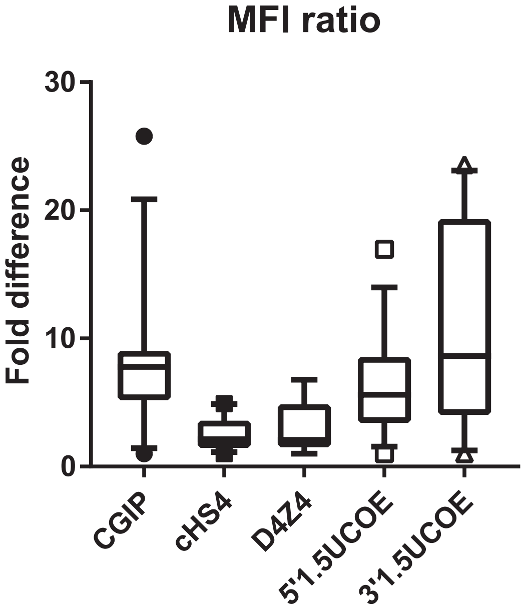

Figure S4. Construct-dependent clonal variation in eGFP expression levels. eGFP-expression MFIs for the clones in each group were normalized to the lowest expressing clone in the individual groups. Boxes are displayed as Q2+Q3 quantile, and whiskers show 10-90 percentile.

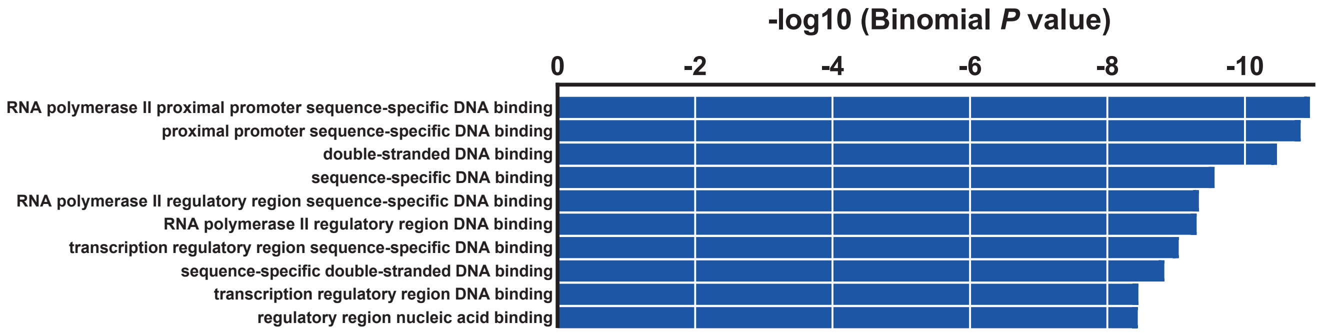

Figure S5. Top ten enriched GO terms for the selected subset.

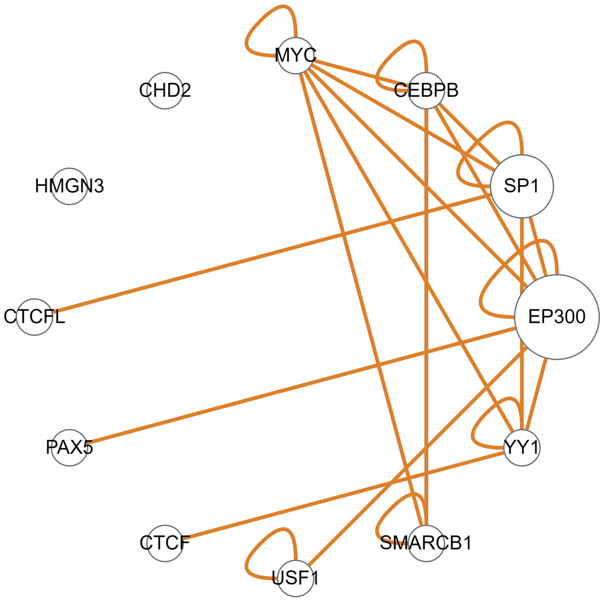

Figure S6. esyN protein-protein interaction network for the selected subset of transcription factors. The size difference indicates the most central nodes as calculated by the betweenness centrality of each node.

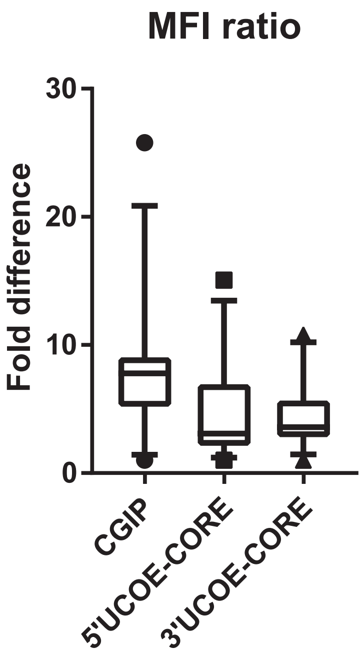

Figure S7. Clonal variation in eGFP expression levels in UCOE-CORE clones. eGFP-expression MFIs for the clones in each group were normalized to the lowest expressing clone in the individual groups. Boxes are displayed as Q2+Q3 quantile, and whiskers show 10-90 percentile.

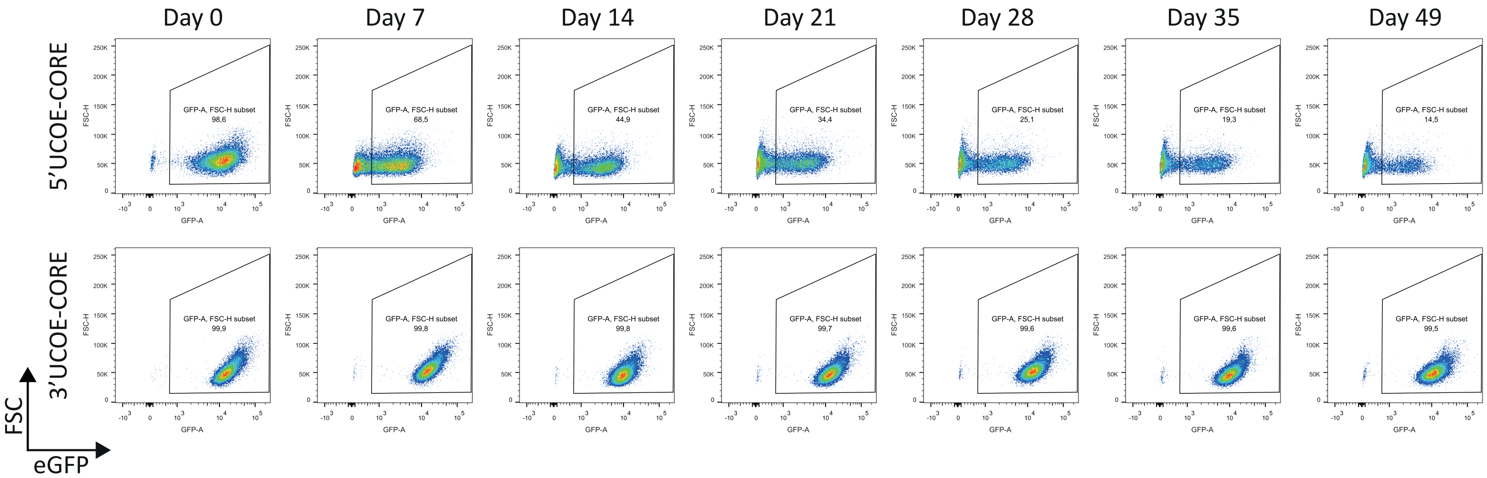

Figure S8. Flow cytometric analysis of representative clones harbouring either the 5'UCOE-CORE or the 3'UCOE-CORE.

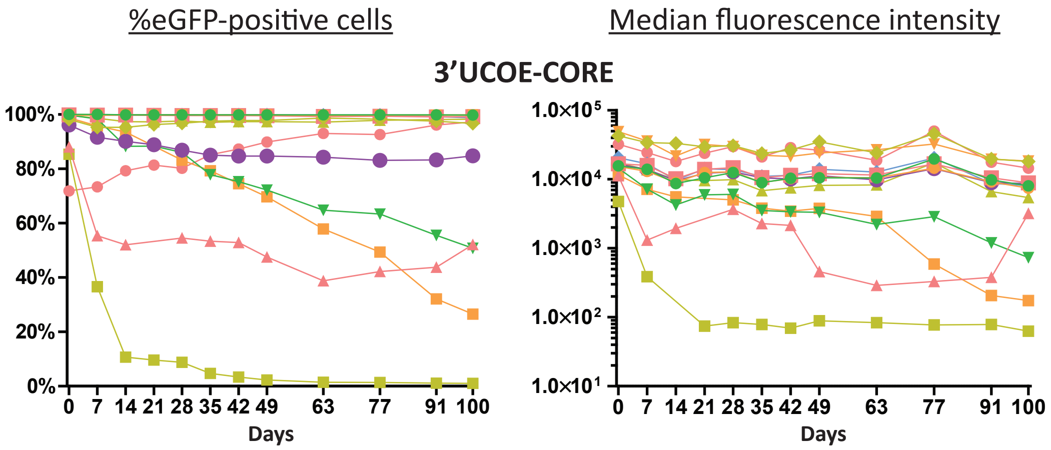

Figure S9. Extended analysis of clones containing the 3'UCOE-CORE. Days 0-49 correspond to data presented in figure 4B.

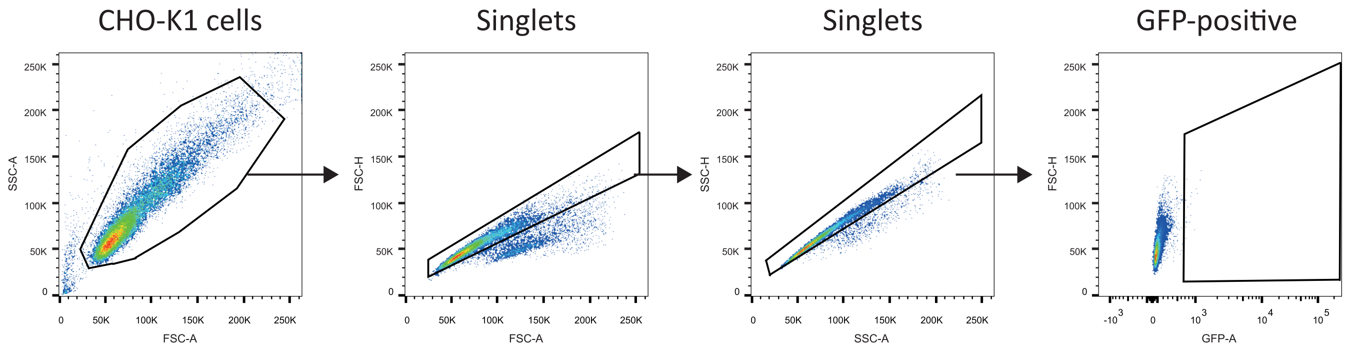

Figure S10. Flow cytometry gating strategy.
