# Supplementary material for: Sustained transgene expression from sleeping beauty DNA transposons containing a core fragment of the HNRPA2B1-CBX3 ubiquitous chromatin opening element (UCOE)
Source: BMC Biotechnol. 2019 Nov 9;19:75. doi: 10.1186/s12896-019-0570-2 (PMC6842454; doi:10.1186/s12896-019-0570-2)
Supplement: Supplementary file 2 — Additional file 2. List of proteins with identified ChIP-seq peaks from the ENCODE project within the CBX3-HNRPA2B1 locus. [file 12896_2019_570_MOESM2_ESM.pdf]

#bin	chrom	chromStart	chromEnd	name	score	expCount	expNums	expScores
785	chr7	26240748	26241128	ARID3A	175	1	422	175
785	chr7	26240385	26240641	ATF1	508	1	468	508
785	chr7	26239563	26240047	ATF3	216	1	45	216
785	chr7	26240140	26240496	BCL3	148	2	83,209	148,119
785	chr7	26239838	26240386	BCLAF1	223	2	84,21	223,22
785	chr7	26240169	26240548	BHLHE40	678	2	284,472	428,678
785	chr7	26240534	26240838	BRCA1	169	1	374	169
785	chr7	26239755	26240300	CCNT2	375	1	476	221
785	chr7	26240014	26240777	CEBPB	276	2	85,477	276,191
785	chr7	26240167	26240443	CEBPD	191	1	173	191
785	chr7	26238382	26240430	CHD1	706	4	4,23,287,344	572,160,157,162
785	chr7	26239624	26240600	CHD2	396	5	288,345,380,427,479	156,239,135,292,310
785	chr7	26240291	26240571	CREB1	731	1	47	731
785	chr7	26240162	26240628	CTCF	278	8	2,267,290,493,603,604,605,628	100,146,117,201,177,261,181,278
785	chr7	26240246	26240416	CTCFL	116	1	214	116
785	chr7	26239297	26240022	E2F1	1000	1	572	315
785	chr7	26239647	26239983	E2F4	156	1	494	156
785	chr7	26239794	26239951	E2F6	267	1	215	267
785	chr7	26241050	26241361	EBF1	1000	2	86,292	1000,1
785	chr7	26239775	26240051	EGR1	214	2	138,216	165,214
785	chr7	26240290	26241031	ELF1	390	4	50,88,175,217	198,299,179,281
785	chr7	26239995	26240477	ELK1	156	2	293,496	156,153
785	chr7	26239535	26240011	ELK4	223	1	388	223
785	chr7	26240151	26240608	EP300	173	2	99,263	135,173
785	chr7	26240221	26240445	ETS1	343	2	89,218	95,343
785	chr7	26240386	26240620	FOSL1	1000	2	139,219	1000,612

785	chr7	26240489	26241009	FOXM1	260	1	90	260
785	chr7	26240272	26240608	FOXP2	176	2	251,255	176,129
785	chr7	26239453	26239993	GABPA	227	1	54	227
785	chr7	26240423	26240690	GTF2B	551	1	499	551
785	chr7	26240147	26240802	GTF2F1	369	3	349,389,500	367,147,369
785	chr7	26239858	26240382	HDAC1	392	1	26	392
785	chr7	26239561	26240057	HMGN3	171	1	501	171
785	chr7	26240252	26240536	HSF1	382	1	433	382
785	chr7	26240335	26240784	IRF1	1000	3	504,505,506	1000,1000,156
785	chr7	26240336	26240646	JUN	126	1	482	126
785	chr7	26239822	26240066	JUND	203	1	142	203
785	chr7	26240132	26240515	KDM5B	1000	1	31	1000
785	chr7	26239653	26240106	MAX	441	3	223,352,576	441,159,236
785	chr7	26240209	26240806	MAZ	263	1	512	215
785	chr7	26240174	26240464	MEF2A	128	1	93	128
785	chr7	26240152	26240448	MEF2C	134	1	94	134
785	chr7	26239626	26240579	MXI1	253	4	298,397,441,513	171,133,253,133
785	chr7	26240472	26240923	MYBL2	776	1	186	776
785	chr7	26239584	26240834	MYC	545	18	347,382,485,486,487,488,489,490,560,561,575,600,614,620,623,624,625,626	169,155,145,119,262,144,515,149,135,198,180,205,159,117,332,293,307,244
785	chr7	26239708	26240304	NFIC	260	1	97	260
785	chr7	26239671	26239981	NR3C1	213	1	58	213
785	chr7	26240161	26240514	PAX5	170	2	101,129	170,139

785	chr7	26240194	26240414	PBX3	155	1	102	155
785	chr7	26239760	26240500	PHF8	1000	1	30	1000
785	chr7	26239974	26240560	PML	1000	2	103,227	1000,95
785	chr7	26238738	26242196	POLR2A	1000	72	11,22,32,41,61,62,78,104,105,123,124,130,131,146,147,163,168,191,192,206,207,228,229,256,259,275,279,283,306,307,308,326,328,330,332,334,336,338,340,362,366,402,403,445,446,447,461,465,520,521,522,523,525,526,527,563,564,577,583,585,596,599,602,610,613,616,619,622,632,633,634,637	841,711,484,811,1000,1000,1000,1000,571,357,487,386,1000,309,510,632,799,365,1000,421,291,201,1000,241,953,275,337,124,304,233,219,449,186,899,593,713,1000,389,1000,108,314,1000,228,160,802,227,300,580,346,257,763,365,770,582,181,287,207,1000,1000,290,294,170,229,259,179,777,111,310,1000,1000,540,397
785	chr7	26239696	26240016	POU2F2	244	1	106	244
785	chr7	26241258	26241474	RAD21	179	1	264	179
785	chr7	26239567	26240491	RBBP5	365	1	33	237

785	chr7	26240375	26240671	RCOR1	263	1	492	263
785	chr7	26240066	26240530	RDBP	170	1	514	170
785	chr7	26239912	26240557	RELA	423	5	300,325,329,331,335	172,240,423,387,181
785	chr7	26239605	26240524	REST	415	9	59,98,144,167,189,226,257,258,274	316,369,227,157,273,317,170,370,239
785	chr7	26240237	26240581	RFX5	189	2	311,406	189,141
785	chr7	26240130	26240500	RUNX3	307	1	109	307
785	chr7	26239738	26240248	SAP30	183	1	34	183
785	chr7	26240435	26240955	SIN3A	184	1	357	184
785	chr7	26239683	26240007	SIN3AK20	119	1	151	119
785	chr7	26240280	26240756	SIX5	276	4	64,111,152,233	256,211,276,253
785	chr7	26240153	26240534	SP1	228	2	112,153	228,144
785	chr7	26240291	26240773	SP4	1000	1	155	1000
785	chr7	26240476	26240860	SPI1	244	2	107,126	195,244
785	chr7	26240218	26240462	SRF	368	2	113,236	368,113
785	chr7	26239960	26240436	STAT1	161	1	410	161
785	chr7	26240925	26241281	STAT2	141	1	540	141
785	chr7	26240353	26240700	STAT3	352	4	566,567,568,569	341,183,204,352
785	chr7	26239772	26240402	STAT5A	339	2	114,237	339,246
785	chr7	26239384	26241650	TAF1	1000	10	65,115,127,132,157,169,199,238,254,261	225,898,961,701,1000,171,192,175,506,261
785	chr7	26240157	26240474	TAF7	840	2	158,239	840,303
785	chr7	26240181	26240691	TBL1XR1	447	3	316,543,544	268,447,225
785	chr7	26239397	26239941	TBP	182	2	317,359	182,152
785	chr7	26239694	26240476	TCF12	347	3	66,116,159	337,347,169

785	chr7	26240240	26240440	TCF3	391	1	117	391
785	chr7	26240227	26240731	TCF7L2	276	2	363,414	276,245
785	chr7	26240939	26241469	TFAP2A	335	1	369	335
785	chr7	26240972	26241488	TFAP2C	597	1	370	597
785	chr7	26240193	26240457	USF1	134	1	243	134
785	chr7	26240088	26240488	WRNIP1	203	1	320	203
785	chr7	26239353	26240190	YY1	1000	13	70,119,128,133,162,164,203,244,245,266,321,551,579	930,1000,944,1000,441,174,402,485,665,578,232,435,308
785	chr7	26240103	26240448	ZBTB33	130	2	120,246	129,13
785	chr7	26239814	26240218	ZBTB7A	169	1	247	169
785	chr7	26241040	26241330	ZNF143	172	1	361	172
785	chr7	26240033	26240629	ZNF263	288	1	368	288
